# Variation of mechanical and thermal properties in sustainable graphene oxide/epoxy composites

**DOI:** 10.1038/s41598-018-34976-6

**Published:** 2018-11-08

**Authors:** Hongran Zhao, Jiheng Ding, Haibin Yu

**Affiliations:** Key Laboratory of Marine Materials and Related Technologies, Zhejiang Key Laboratory of Marine Materials and Protective Technologies, Ningbo Institute of Materials Technology and Engineering, Chinese Academy of Sciences, Ningbo, 315201 China

## Abstract

In this work, the functional graphene oxide (bGO) was facilely synthesized through a grafted reaction between graphene oxide (GO) and bio-based bis-furan di-epoxide (BFDE). The structure of bGO was confirmed by FTIR spectra and Raman spectra. The properties of polymer composite materials depend on the distribution of the nanofiller in the matrix and due to the presence of polymer chains our bGO sheets exhibit a better dispersibility in solvents and polymer matrix, which provides a potential opportunity for the preparation of BFDE composites with excellent performance. Bio-based BFDE composites containing 0.05–0.5 wt.% of bGO exhibit superior mechanical and thermal properties. The addition of just 0.5 wt% such bGO to an BFDE causes 80%, 49%, 21%, 69% and 97% enhancement in tensile strength, flexural strength, flexural modulus, critical stress intensity factor and critical strain energy release rate, respectively. The thermal decomposition temperature *T*_d_ of bGO/BFDE composites was increased about ~17 °C compared to blank BFDE sample. In addition, we found that introducing unmodified GO to epoxy matrix lead to an insignificant increase of the thermal property of the resulting GO/BFDE composites. The enhanced mechanical properties and thermal properties of bGO/BFDE composites could be attributed to strong interfacial interactions and high affinity between bGO and epoxy matrix.

## Introduction

Ever since it was discovered, graphene (G), a single-layered two-dimensional structure, has attracted wide attention due to its ultrahigh thermal conductivity and electric conductivity^1–3^, large specific surface and high Young’s modulus^4–6^. It has been considered as useful nanofiller in polymer composites for the enhanced properties, and the small amount addition of G can improve the properties of the composites^4^. Generally, G is prone to irreversible aggregation and precipitation in water and organic media due to the insolubility of itself and the existence of Van der Waals forces and the π-π stacking between the lamellae, which greatly limits many practical applications of G^7^. Graphene oxide (GO) has a similar spatial two-dimensional structure, and different types of functional groups such as hydroxyl, epoxides, and carbonyl groups distributed on the surface of GO plane^8^. Besides, GO is more readily available than G, which has a greater range of applications and frequency of utilization at present^9–12^, for instance, Hummers method is a common, simple and economic approach for obtaining graphene oxide. However, in any case, the polar groups on the surface of GO have extremely negative effects on the compatibility with the resin matrix, which has a negative impact on the properties of polymer composites. As a result, surface modification of GO becomes one of the necessary methods. In terms of many functional modifications of GO, graft of polymer on GO has a unique fine effect. In previous work, Lin *et al*.^13^ prepared a polyethylene (PE) grafted GO/polyethylene composite (PE-g-GO/PE), the research revealed the PE-g-GO with good dispersion in the polymer matrix, and the polymer composites were significantly enhanced in terms of stiffness and strength. Lai *et al*.^14^ reported that diglycidyl ether of bisphenol-A functionalized GO (DGEBA-f-GO), and found the DGEBA-f-GO effectively enhanced the compatibility with epoxy matrix, resulting in higher Young’s modulus and tensile strength than either the pure epoxy or the GO/epoxy composites. Zheng *et al*.^15^ reported the similar research in the modified GO with Lai *et al*. Then the modified GO was embedded into the polycarbonate (PC), finding that the interfacial interaction between polymer matrix and GO was enhanced owing to the unreacted epoxide groups and hydroxyl groups in the grafted epoxy chain, which could further react with PC to form a chemical bond, resulting in remarkably enhancing the mechanical properties of the composites. Although the functional GO modified by polymer chains shows outstanding effect and good application prospect, the petroleum based polymers used are nonrenewable resources and do not meet the requirements of the sustainable development of modern society^16,17^.

**Figure 1:**
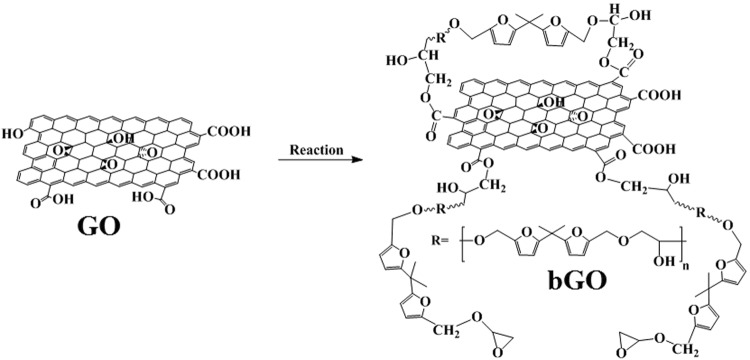
Synthesis of functionalised GO via covalent reaction between BFDE and GO.

In recent decades, more and more attention has been paid to the bio-based materials because of the diminishing petroleum oil reserves and serious environment pollution^14,18^. In this study, a bio-based bis-furan di-epoxide resin (BFDE) was synthesized from renewable furan-2,5-dicarboxylicacid, and we demonstrated the surface functionalization of GO nanosheets (bGO) with BFDE. As shown in Fig. 1, the reaction between BFDE and GO nanosheets occurs mainly between the epoxy group of BFDE and the carboxyl group of GO. Due to the high reaction activity between the epoxides and carboxyl groups, the reaction can be readily occurred under simple conditions. The mechanical and thermal properties of BFDE composites containing different content of bGO were investigated. The objective of this work is to obtain novel bio-based epoxy composites with superior properties to replace the traditional petroleum-based DGEBA resin. Up to now, such the bio-based epoxy composites have never been reported.

## Experimental Section

### Functionalization of GO sheets

Functionalization of bGO was conducted according to the literatures^14,15^ with some improvements: 100 mg GO powder was uniformly dispersed into acetone by simple sonication and 10 g BFDE was added. Then, 500 mg 20 wt% aqueous NaOH solution was added into the mixture as a catalyst. The mixture was kept at 75 °C for 8 h and stirred under inert atmosphere. After that, as-obtained product was washed with acetone and distilled water for several times to remove uncreated materials. The functionalized GO hereafter denoted as bGO, was then dried at 60 °C for 24 h in vacuum oven. The synthesis method is illustrated in Fig. 1.

### Composites preparation

The bGO/BFDE composites were fabricated by the following procedures. Firstly, bGO sheets were dispersed in acetone (~5 mg/mL) by using a tip sonication (200 W, 50 Hz). Then, the BFDE were added into the bGO acetone solution and ultrasonicated for 30 min in an ice/water bath. The acetone was removed through a constantly degassed treatment in a vacuum oven at 50 °C for 10 min. The curing agent was then added (epoxy and curing agent were 1: 0.8 equivalent ratio) and the mixture was degassed with stirring under vacuum at room temperature for 5 min. To fabricate nanocomposite samples, the mixture was powered into a Teflon mold with cavity dimensions of 3 mm × 10 mm × 60 mm. The curing reaction was performed at 80 °C for 4 h, 30 °C for 24 h. After cooled to room temperature, the cured specimens were used for the property tests. The samples with different content of bGO were named as bGO_0_, bGO_0.05_, bGO_0.1_, bGO_0.3_, and bGO_0.5_, respectively. For example, bGO_0.05_ represents the BFDE composite containing 0.05 wt% bGO.

### Measurements and Characterization

FTIR measurements of the samples were obtained on a Nicolet 6700 spectrometer (Thermo Fisher Scientific, Inc., Waltham, MA, USA). Note that the free BFDE physically adsorbed on GO sheets was removed by strict washing prior to the tests. The thermogravimetric analysis (TGA) was investigated using PerkinElmer Pyris Diamond TG/DTA instrument in the temperature ranging from 25 to 600 °C at a heating rate of 10 °C/min under nitrogen flow (200 mL/min), and the initial weight of each sample tested was approximately 5 mg. Raman spectra was recorded with Renishaw Via Reflex spectrometer. The morphologies of GO and bGO sheets were analyzed by a transmission electron microscopy (TEM, FEI Tecnai F20) and a Scanning Probe Microscope (AFM, Vecco Dimension 3100). X-ray diffraction (XRD) was conducted by a BRUKER AXS D8 ADVANCE X-ray diffractor. The differential scanning calorimetry (DSC) of the curing system was studied using a Netzsch DSC 214 Polyma instrument in the temperature ranging from 0 to 180 °C at a heating rate of 10 °C/min under nitrogen atmosphere according to ISO 11357:2013, and samples (around 10 mg) were sealed in hermetic aluminium pans. For each specimen, two temperature ramps were performed to determine the glass transition temperature (T_g_), and then the T_g_ value was determined during the second temperature scan. Scanning electron microscopy (SEM; FEI Quanta FEG 250) was employed to observe the fracture morphology. Dynamic thermomechanical analysis (DMA) was analyzed in tensile mode by a Dynamic Mechanical Analyzer (METTLER TOLEDO, DMA/SDTA861e), which was performed at 1 Hz with a heating rate of 3 K/min and with working temperature between 25 and 130 °C. The tensile test and bending test were conducted using a microcomputer control electronic universal testing machine (Instron 5567) according to ASTM D638 and ASTM D790, respectively. The impact test was carried out by a simple beam-cantilever beam combined impact testing machine (XJ-50Z) according to ISO 180:2000.

## Result and Discussion

### Characterization of bGO

**Figure 2:**
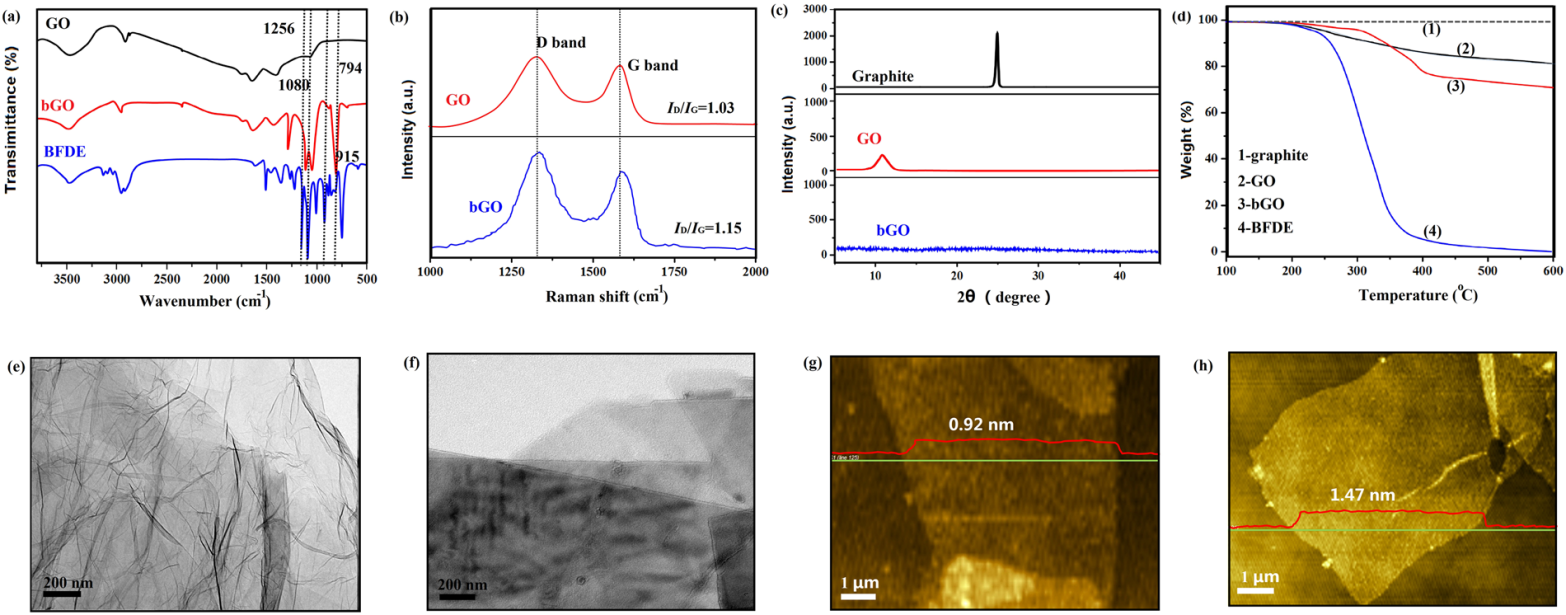
Structural and morphological characterization: (**a**) FTIR spectra, (**b**) Raman spectra, (**c**) XRD spectra, and (**d**) TGA curves. TEM images of GO (**e**) and bGO (**f**). AFM images of GO (**g**) and bGO (**h**).

The evidence for the covalent reaction between GO sheets and BFDE was confirmed by FTIR spectra (Fig. 2a). In the FTIR spectrum of the bGO sample, the two characteristic absorption peaks at 795 cm^−1^ and 1080 cm^−1^ were contributed to the furan ring and ether groups in BFDE, respectively, implying the presence of the grafted BFDE chains on bGO. Especially, the characteristic absorption at 1256 cm^−1^ was related to C-O-C, which provided direct evidence for the forming in the reaction between bGO and BFDE. Moreover, the peak at 1725 cm^−1^ corresponding to C=O of -COOH on GO shifted to 1720 cm^−1^ owing to the formation of C-O bond in bGO. In addition, the peak at 915 cm^−1^ of bGO disappeared, which also indicated the forming reaction between BFDE and GO^19^.

The structure of bGO was further investigated by Raman spectroscopy as shown in Fig. 2b. The Raman spectrum of GO showed a D band at 1320 cm^−1^ and a G band at 1580 cm^−1^, which were attributed to the first order zone boundary phonon mode connected with defects in the surfaces or edges and the radial C-C stretching mode connected with sp^2^ bond carbon atoms individually^20^. Similar Raman spectrum of was observed in bGO. Generally, *I*_D_/*I*_G_ value is opposite to the average size of sp^2^ domains^21^, the larger value of *I*_D_/*I*_G_, the smaller the uniformity of the carbon crystal structure is. The higher *I*_D_/*I*_G_ value (1.03) of GO confirmed the deformation of the C-C bond and the destruction of the lattice symmetry in GO due to the reduction in the average size of the in-plane sp^2^ domain attributed to the oxidation. After modification, a slight increase in *I*_*D*_/*I*_*G*_ value (1.15) was observed in bGO, which confirmed the successful attachment of functional groups on bGO nanosheets. Raman spectra also implied that the structure of bGO lattice was not damaged during the reaction. Figure 2c showed XRD patterns of the graphite, GO and bGO. The sharp diffraction peak at 26.5° of graphite was the (002) reflection peak, suggesting the graphite plane composed of well ordered graphenes with an interlayer spacing of 3.35 Å. A diffraction peak at ~10.8° was observed in GO, which was related to the existence of functional groups on the surface of GO^22^. For the XRD spectrum of bGO, no clearly visible peak can be observed, indicating the sheets stack together in a looser way caused the decreased size of sp^2^ bonded crystalline domains after BFDE functionalization^23^. TGA is a fairly simple method to analysis the thermal stability and the presence of functional group in bGO. As shown in Fig. 2d, graphite is a thermally stable material with no weight loss over the entire testing temperature range. For GO, a major weight loss stage at 200–300 °C was observed due to the pyrolysis of oxygen-containing functional groups on the sheets, yielding CO_2_, CO and H_2_O vapors, and ~15% weight loss at 600 °C. Two major weight loss stages at 150–190 °C and 300–400 °C in the bGO’s TGA curve were seen. For the first stage, which was probably attributed to the decomposition of BFDE chains grafted on bGO sheet; and for the second stage, which was the result of the decomposition of oxygen-containing functional groups at high temperature. Moreover, in consideration of the residue after the first pyrolysis stage, the content of grafted BFDE in bGO was about 8.5%.

The microscopic morphology of bGO was characterized by AFM and TEM. Before testing, GO and bGO were dispersed into acetone by ultrasonication, and then deposited onto a copper grid and a freshly cleaved mica surface, respectively. As shown in the Fig. 2e, the GO showed a transparent clean surface with thin ripples. The ripples were an inherent property of graphene nanosheets, and it’s on account of the extra thermodynamic stability of two-dimensional membranes caused by microscopic crumpling^3^. The surface of bGO seemed to be covered by a thin BFDE layer (in Fig. 2f), which was similar with the GO structure reported refs^24,25^. Figure 2(g,h) showed the representative AFM images of GO and bGO sheets. The average thickness of GO was about 0.92 nm as observed in Fig. 2g. After functionalization, the bGO presented an increase average thickness to ~1.47 nm, indicating the variation of bGO surface morphology caused by the surface functionalization. Interestingly, there was also a color change from brown GO to black bGO (Fig. S3). The successful functionalization of bGO was further confirmed by the change of the solubility of bGO in organic solvents. We prepared CH_2_Cl_2_ dispersions of GO and bGO at a concentration of 0.1 mg/mL. The bGO dispersion was stable and no obvious precipitation was observed after 1 month of storage. However, direct dispersion of GO in CH_2_Cl_2_ result in agglomerates and precipitation after only several days. The results revealed that the conversion of the hydrophilic GO to the hydrophobic bGO. In order to exclude the possible affection of the physically adsorbed BFDE chains on the dispersibility of bGO, the mixture of GO and BFDE was dispersed into CH_2_Cl_2_. As predicted, GO sheets were completely precipitated in CH_2_Cl_2_, indictaing the dispersibility is the result of chemical modification BFDE instead of the physically adsorbed BFDE.

### Dispersion of bGO in polymer matrix

**Figure 3:**
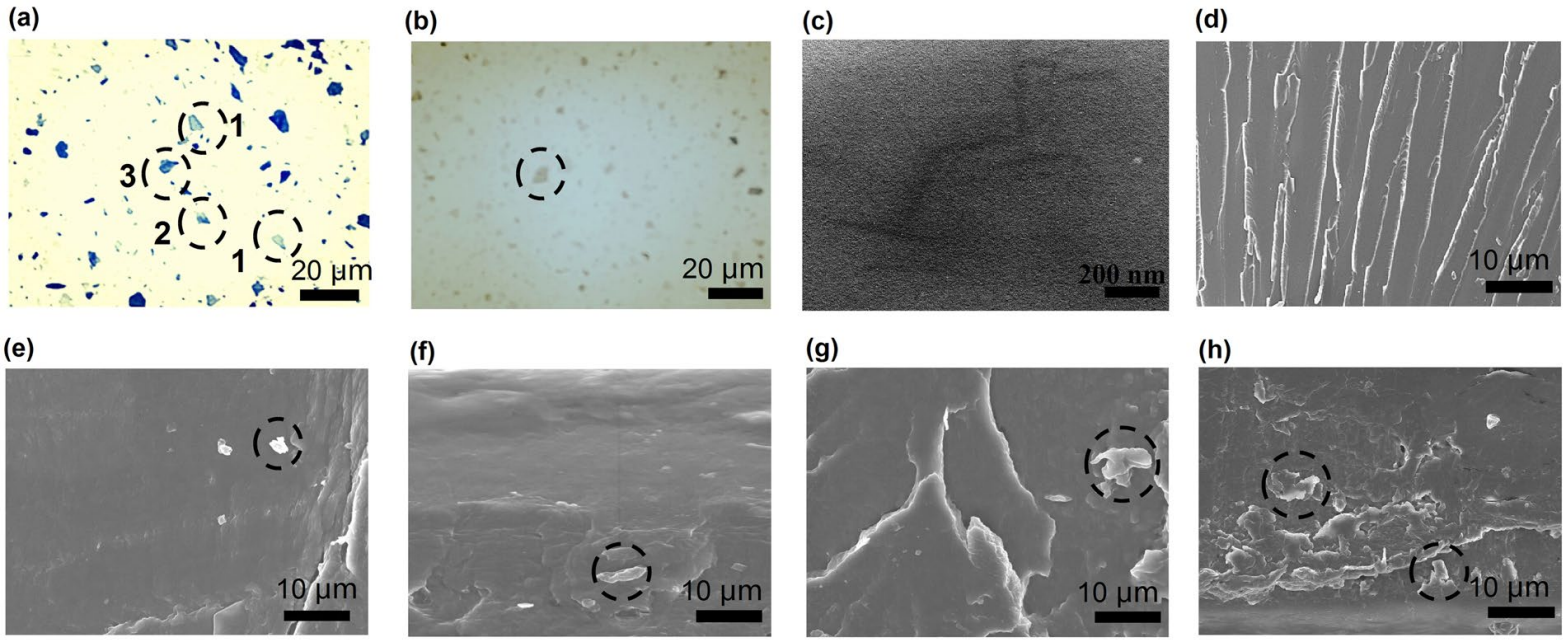
Characterization of composites: the optical micrographs of the liquid epoxy mixtures containing bGO before (**a**) and after (**b**) curing; TEM of bGO/BFDE composite with 0.5% weight content of bGO (**c**); SEM micrographs of bGO/BFDE composite with bGO_0_ (**d**), bGO_0.05_ (**e**), bGO_0.1_ (**f**), bGO_0.3_ (**g**), and bGO_0.5_ (**h**) weight content of bGO.

The bGO/BFDE colloid solution was prepared by adding bGO into BFDE matrix, blending until uniform distribution of bGO in epoxy resin. Addition of the uniform and transparent bGO into the transparent BFDE matrix, the bGO/BFDE mixtures exhibit a fully homogeneous and transparent nature and evident Tyndall effect (Fig. S4). This was another evidence for the uniform distribution of bGO in the polymer matrix. Figure 3(a,b) displays the optical microscopy images of uncured bGO/BFDE solutions before and after curing. There were numerous bGO sheets seen within the acquisition spot. All of the bGO sheets, except the one placed in the circle, had similar opacity, the sheets were 1, 2, and 3 layers. Figure 3b clearly showed the difference in the contrast (opacity) of bGO sheets in epoxy matrix after curing, indicating that most of the bGO sheets were fully dispersed condition. In order to further determine the dispersion of GO in matrix, TEM was used to observe the ultrathin sections of the bGO_0.5_ composites. As shown in Fig. 3c, bGO sheets were relatively evenly dispersed in the matrix, and no large aggregates could be seen, which suggested that bGO sheets remained good dispersed conditions in the polymer composites. Typical cross-sectional view SEM images of epoxy composites with different bGO loadings were shown in Fig. 3(d–h). All as-fabricated epoxy composites were prepared by a simply solution-casting procedure. It can be seen that bGO_0_ samples showed a relatively homogeneous morphology with sidestep-like structure in Fig. 3d. Obviously, the fractured surfaces of the bGO/BFDE composites were rougher compared with that of blank bGO_0_ sample. The bGO sheets were homogenously dispersed and embedded in epoxy matrix, as shown in Fig. 3(e–h), suggesting good compatibility between the bGO and polymer matrix. The debonding and wrinkled bGO sheets were careful observed in Fig. 3(e–h). With the increase of bGO sheets, the surface became rougher and bumpier and more dimples appeared, showing the improved toughness of the material, which might result in improving tensile properties and bending properties^26,27^.

### Mechanical and Thermal Properties of bGO/BFDE composites

**Figure 4:**
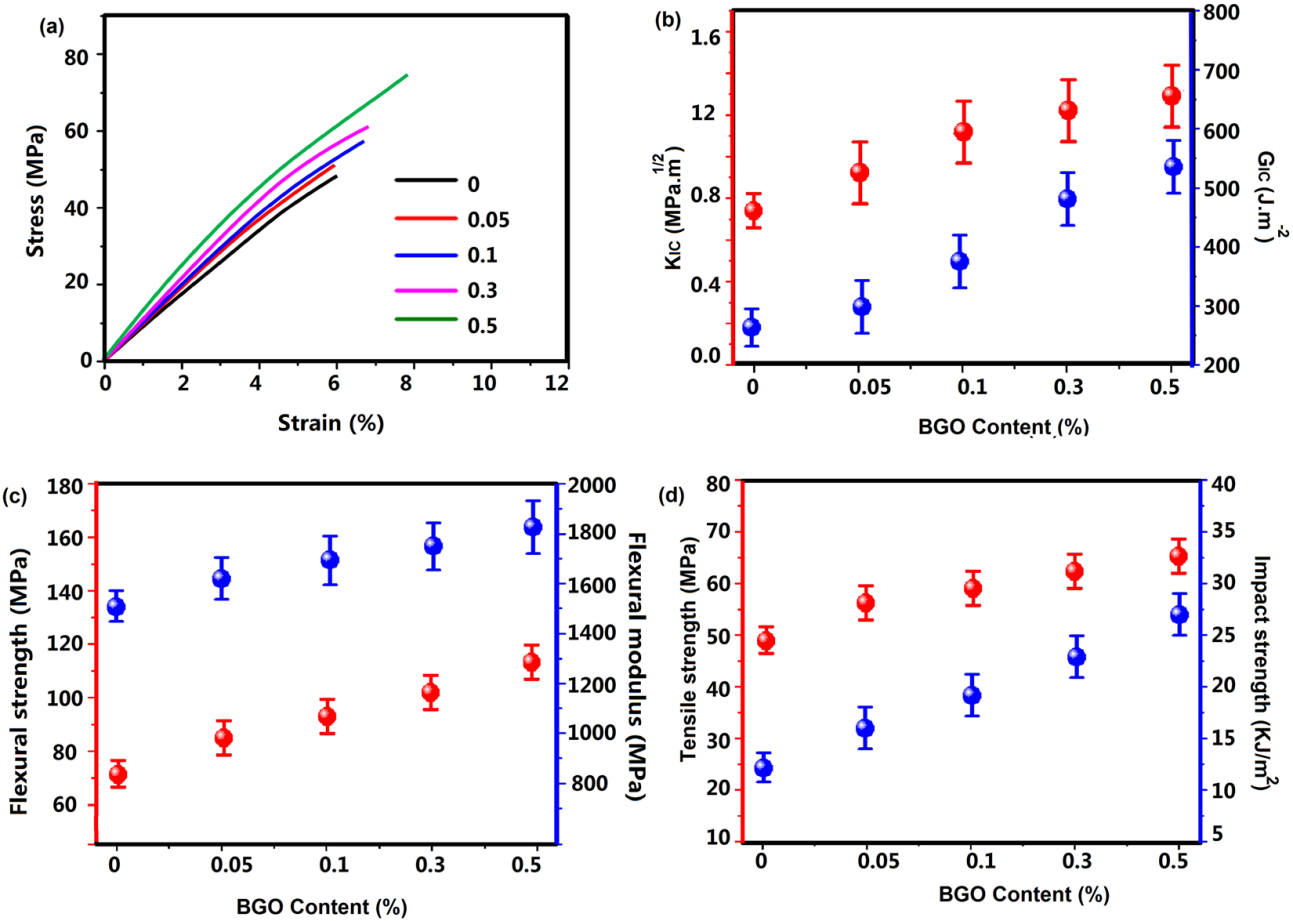
Mechanical and thermal properties of bGO/BFDE composites with various weight fractions of bGO: (**a**) Tensile strength, (**b**) fracture toughness, (**c**) flexural modulus and ultimate flexural strength, and (**d**) impact strength.

**Figure 5:**
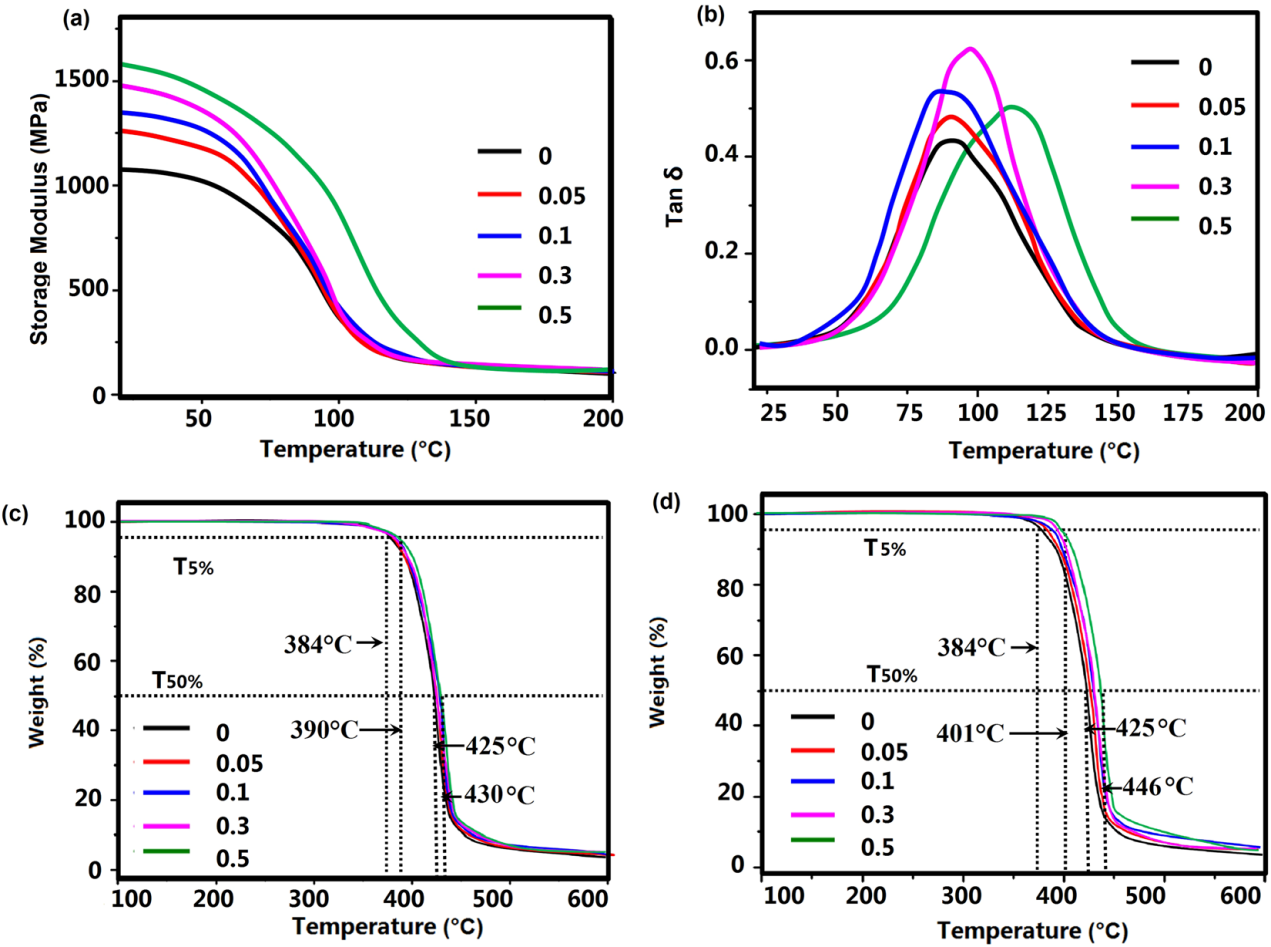
Dynamic mechanical properties of bGO/BFDE composite (**a**,**b**); TGA curves of neat BDFE epoxy (**c**) and bGO/BFDE composite (**d**).

Monotonic material performance of the bGO based nanocomposites was summarized in Fig. 4 and Table 1. It is well known that a small amount of uniformly dispersed graphene based materials will increase polymer mechanical properties significantly. Figure 4a showed that the tensile properties were increased greatly with the addition of bGO. Higher loadings of bGO displayed increased tensile stiffness when compared to blank BFDE sample. Ultimate tensile strength showed maximum improvement about ~80% in composite with 0.5 wt % of bGO. Compared with neat BFDE (Fig. 4b), the bGO/BFDE composites showed 21%, 56%, 61% and 69% increase in critical stress intensity factor (K_IC_), 12%, 40%, 81% and 97% increase in critical strain energy release rate (G_IC_), respectively. Similarly, flexural strength and modulus (Fig. 5c) saw the largest incremental increase at the lowest bGO concentration, 21% and 51%, respectively, at 0.5 wt % bGO. Compared with blank bGO_0_ sample, the bGO_0.05_, bGO_0.1_, bGO_0.3_ and bGO_0.5_ composites showed 37%, 56%, 87% and 130% increase in impact strength (Fig. 4d), respectively.

**Table 1:** Thermal and mechanical properties of bGO/BFDE composites.

BFDE-GO Content	Tensile strength/MPa	K_IC_ (MPa m^1/2^)	G_IC_ (J/m^2^)	Flexural strength MPa	Flexural modulus MPa	Impact strength (kJ/m^2^)
0 wt.%	43.0 ± 1.1	0.72 ± 0.08	267 ± 23	71.8 ± 3.2	1525 ± 21	12.0 ± 0.5
0.05 wt.%	47.1 ± 0.4	0.87 ± 0.11	298 ± 31	87.1 ± 1.5	1623 ± 27	16.4 ± 1.2
0.1 wt.%	58.2 ± 1.3	1.12 ± 0.14	375 ± 40	92.3 ± 2.0	1700 ± 33	18.7 ± 1.5
0.3 wt.%	63.1 ± 2.2	1.16 ± 0.15	482 ± 37	101.1 ± 2.7	1742 ± 45	22.4 ± 1.8
0.5 wt.%	77.3 ± 2.4	1.22 ± 0.15	527 ± 41	108.2 ± 1.9	1838 ± 51	27.6 ± 1.9

In Fig. 5a, dynamic mechanical analysis provided information on storage modulus of bGO/BFDE composites, which showed a trend of decreasing with the increase of test temperature due to the softening of polymer chains at high temperature^10^. In addition, the storage modulus of bGO_0.5_ increased by ~48% up to the highest of 1625 MPa compared to bGO_0_ sample (1100 MPa). In the glassy plateau, the polymer chains and segments were “frozen”, and addition of bGO sheets into epoxy resin increased the storage modulus over the whole temperature range, this may owe to bGO reinforcement and the better interfacial interactions between bGO and the matrix due to the improvement of the interfacial compatibility between the sheets and the epoxy matrix caused by BFDE chains. Therefore, it can be concluded that bGO could enhance mechanical properties of bGO/BFDE composites, which might because improved dispersion and exfoliation properties of the nanosheets as well as strong interfacial bonding were beneficial for enhancing the load transfer from the substrate to the lamellae after grafting of bGO on BFDE, which significantly increased the mechanical properties.

The thermal stability of cured BFDE composites with different content of bGO was analyzed using loss factor (tan delta) and TGA. The glass transition temperature (*T*_g_) was recorded based on a maximum peak of loss factor curve. As shown in Fig. 5b, the *T*_g_ value as-obtained from tan delta of the composites showed a clearly increase trend from 90.3 °C to 116.8 °C with increase of bGO. The reason of increased *T*_g_ value was speculated that wrinkled morphology of bGO sheets with high specific surface constrain the segmental movement of polymer chains to a certain degree. Figure 5(c,d) showed TGA thermograms of cured bGO/BFDE composites with various bGO loads. For comparison purposes, the TGA thermograms of bGO epoxy composites containing different unmodified GO sheets were tested too. It can be seen that the main weight degradation behavior took place at around 300–500 °C, which was attributed to the degradation of epoxy network. From the Fig. 5c, the decomposition temperature *T*_d_ (5% weight loss) of epoxy composites increased by ~6 °C, and the unmodified GO sheets loading did not appreciably affect the *T*_d_ value of epoxy composites. For the bGO/BFDE composites in Fig. 5d, the *T*_d_ value showed an increased trend with the increase of bGO content, and 0.5 wt% of bGO resulted in a significant increase of ~17 °C. In addition, the *T*_50%_ (50% weight loss) of bGO/BFDE composites also showed an increased trend with the increase of bGO content, and 0.50 wt% sheets resulted in a significant increase of 21 °C, while the incensement of *T*_50%_ for the GO/BFDE was only ~5 °C. The enhanced thermal stability of bGO/BFDE composites was mainly attributed to the high heat capacity of BDFE and a better barrier effect of bGO which retarded the volatilization of polymer decomposition products due to a better dispersibility and interface between bGO and epoxy matrix^28–30^.

According to the above mechanical and thermal properties of bio-based BFDE composites containing different content of functionalized GO sheets, we further compared the data of tensile strength, and K_IC_ and T_g_ of various commercial DGEBA systems and the relative increments after embedded initial or modified GO sheets. In order to obtain the related values with this work, the epoxy systems chosen all have the same GO mass content of 0.50%. From Table 2, although the tensile strength and fracture toughness as well as thermal properties were relatively lower compared with commercial epoxy systems, the increments of properties were much higher than those of commercial epoxy systems, indicating that bGO was much more effective than other functionalized GO in enhancing mechanical and thermal properties of epoxy systems. It is well known that the mechanical and thermal properties of epoxy composites were closely related to the dispersibility, aspect ratio of GO nanosheets, and testing conditions, etc. As a result, the bGO/BFDE systems had the comparable mechanical and thermal properties with that of commercial epoxy composites in previous studies, as summarized in Table 2.

**Table 2:** Comparison of increments in the *T*_*g*_, tensile strength, *K*_*IC*_ among various commercial DGEBA composites containing 0.5 wt.% GO nanosheets.

Ref.	Tensile strength/% (Matrix)	K_IC_/% (Matrix)	T_g_/°C (Matrix)
^1^	—	—	2 (150)
^20^	1 (70)	14 (1.4)	—
^27^	11 (69.7)	25 (1.02)	—
^27^	12 (69.7)	37 (1.02)	—
^5^	12 (65)	60 (0.75)	3.0 (126.3)
^27^	13 (69.7)	26 (1.02)	—
^19^	22 (52.98)	25 (0.503)	3.9 (~147.9)
^30^	60 (2.5)	—	1.0 (62.0)
^19^	61 (52.98)	33 (0.503)	1.6 (~147.9)
Our work	79 (43.00)	69 (0.72)	26.5 (~90.3)

Based on the above analysis, we study the mechanical and thermal properties of epoxy composites including flexural strength, flexural modulus, storage modulus, glass transition temperature (*T*_g_), decomposition temperature (*T*_d_) and TGA curves of various bGO/BFDE composite systems. It can be concluded that the bGO/BFDE composites with good balance among the outstanding mechanical and thermal properties at a low content of sheets (≤0.5 wt %), indicating the bGO sheets could improve the related performance of BDFE composites. We still believe that the bio-based bGO/BFDE system is expected to become a substitute for the traditional petroleum DGEBA systems in the future.

## Conclusion

In summary, we provide a method for introducing bio-based BFDE into the GO surface, affording uniform distribution of bGO in the BFDE matrix. The transparent appearance of as-prepared bGO/BFDE system, the optical microscopy of uncured bGO/BFDE system confirmed that homogeneous dispersion of bGO in the epoxy matrix. The bGO/BFDE with 0.05–0.5 wt % bGO sheets showed increased mechanical and thermal properties. The addition of only 0.5 wt% such BFDE-GO to an epoxy resin gives 80%, 49%, 21%, 69% and 97% enhancement in tensile strength, flexural strength, flexural modulus, critical stress intensity factor and critical strain energy release rate, respectively. The thermal decomposition temperature of bGO/BFDE composites was increased by increase of ~17 °C compared to blank epoxy sample. We believe that the modified bGO prepared by low-cost and bio-renewable BFDE has a great potential for applications in functional fillers for polymer composites.

## Electronic supplementary material

Supplementary Information

## References

[1] Liu Q (2012). Mechanical and thermal properties of epoxy resin nanocomposites reinforced with graphene oxide. Polym. Plast. Technol. Eng..

[2] Posudievsky OY, Papakin MS, Boiko OP, Koshechko VG, Pokhodenko VD (2015). Enhanced and tunable photoluminescence of polyphenylenevinylenes confined in nanocomposite films. Nanoscale Res. Lett..

[3] Amirova L (2017). Homogeneous Liquid phase transfer of graphene oxide into epoxy resins. ACS Appl. Mater. Interfaces..

[4] Ambrosi A, Chua CK, Latiff NM (2016). Graphene and its electrochemistry. Chem. Soc. Rev..

[5] Bortz DR, Heras EG, Martin-Gullon I (2012). Impressive fatigue life and fracture toughness improvements in graphene oxide/epoxy composites. Macromolecules..

[6] Guadagno L (2017). Influence of carbon nanoparticles/epoxy matrix interaction on mechanical, electrical and transport properties of structural advanced materials. Nanotechnology..

[7] Ding JH, Rahman OU, Wang QL, Peng WJ, Yu HB (2017). Sustainable graphene suspensions: a reactive diluent for epoxy composite valorization. ACS Sustain. Chem. Eng..

[8] Vasileiou AA, Kontopoulou M, Docoslis A (2014). A noncovalent compatibilization approach to improve the filler dispersion and properties of polyethylene/graphene composites. ACS Appl. Mater. Inter..

[9] Zhang C, Wu H, Kessler MR (2015). High bio-content polyurethane composites with urethane modified lignin as filler. Polymer..

[10] Gudarzi, M. M., Aboutalebi, S. H., & Sharif, F. In *Graphene Oxide: Fundamentals and Applications*; Dimiev, A. M., Eigler, S. Eds; Wiley: London; Chapter 10, pp 314–365 (2017).

[11] Zhu S, Luo F, Chen WC, Zhu B, Wang GX (2017). Toxicity evaluation of graphene oxide on cysts and three larval stages of Artemia salina. Sci Total Environ..

[12] Park YT (2015). Epoxy toughening with low graphene loading. Adv. Funct. Mater..

[13] Lin Y, Jin J, Song M (2011). Preparation and characterisation of covalent polymer functionalized graphene oxide. J. Mate. Chem..

[14] Wan YJ (2014). Grafting of epoxy chains onto graphene oxide for epoxy composites with improved mechanical and thermal properties. Carbon..

[15] Shen B, Zhai W, Tao M, Lu D, Zheng W (2013). Chemical functionalization of graphene oxide toward the tailoring of the interface in polymer composites. Compos. Sci. Technol..

[16] Umer R, Li Y, Dong Y, Haroosh HJ, Liao K (2015). The Effect of graphene oxide (GO) nanoparticles on the processing of epoxy/glass fiber composites using resin infusion. Int. J. Adv. Manuf. Technol..

[17] Liu F, Wu L, Song Y, Xia W, Guo K (2015). Effect of molecular chain length on the properties of amine-functionalized graphene oxide nanosheets/epoxy resins nanocomposites. RSC Adv..

[18] Jiang T, Kuila T, Kim NH, Ku BC, Lee JH (2013). Enhanced mechanical properties of silanized silica nanoparticle attached graphene oxide/epoxy composites. Compos Sci Technol..

[19] Ding JH, Peng WJ, Luo T, Hai BY (2017). Study on the curing reaction kinetics of a novel epoxy system. RSC Adv..

[20] Bourgeat-Lami E, Faucheu J, Noel A (2015). Latex routes to graphene-based nanocomposites. Polym. Chem..

[21] Kumar P, Maiti UN, Lee KE, Kim SO (2014). Rheological properties of graphene oxide liquid crystal. Carbon..

[22] Ding JH, Zhao HR, Yu HB (2018). A water-based green approach to large-scale production of aqueous compatible graphene nanoplatelets. Sci Rep-uk..

[23] McAllister MJ (2007). Single sheet functionalized graphene by oxidation and thermal expansion of graphite. Chem Mater.

[24] Wang R (2013). Attapulgite-graphene oxide hybrids as thermal and mechanical reinforcements for epoxy composites. Compos Sci Technol..

[25] Cao YW, Lai ZL, Feng JC, Wu PY (2011). Graphene oxide sheets covalently functionalized with block copolymers via click chemistry as reinforcing fillers. J. Mater. Chem..

[26] Liu Q (2012). Mechanical and thermal properties of epoxy resin nanocomposites reinforced with graphene oxide. Polym. Plast. Technol. Eng..

[27] Yousefi N (2013). Simultaneous *in situ* reduction, self-alignment and covalent bonding in graphene oxide/epoxy composites. Carbon..

[28] Schniepp HC, Li JL, Mcallister MJ (2006). Functionalized Single Graphene Sheets Derived from Splitting Graphite Oxide. J. Phys. Chem. B..

[29] Dikin Dmitriy A, Stankovich S, Zimney EJ (2017). Preparation and characterization of graphene oxide paper. Nature..

[30] Gudarzi MM, Sharif F (2012). Enhancement of dispersion and bonding of graphenepolymer through wet transfer of functionalized graphene oxide. eXPRESS Polym Lett..

