# Supplementary material for: Variation of mechanical and thermal properties in sustainable graphene oxide/epoxy composites
Source: Sci Rep. 2018 Nov 8;8:16560. doi: 10.1038/s41598-018-34976-6 (PMC6224379; doi:10.1038/s41598-018-34976-6)
Supplement: Supplementary file 1 — Supplementary Information [file 41598_2018_34976_MOESM1_ESM.doc]

**SUPPORTING INFORMATION**

**Variation of mechanical and thermal properties in sustainable graphene oxide/epoxy composites**

Hongran zhao^1^, Jiheng Ding^1^, Haibin Yu**[[1]](#footnote-2)***

*Key Laboratory of Marine Materials and Related Technologies, Zhejiang Key Laboratory of Marine Materials and Protective Technologies, Ningbo Institute of Materials Technology and Engineering, Chinese Academy of Sciences, Ningbo 315201, China.*

***^1^ These authors contributed equally to this work.***

****Corresponding author, Electronic mail: haibinyu@nimte.ac.cn.***

**Experimental section**

**Materials**

FCA (98%), epichlorohydrin, tetrabutylammonium bromide (TBABr, 99%), lithium aluminum hydride (LiAlH_4_), sodium hydroxide (NaOH), sulfuric acid (H_2_SO_4_), nitric acid, hydrochloric acid (HCl), acetone, and solvents including methanol, ethyl acetate, hexane, tetrahydrofuran (THF), dichloromethane (DCM), graphite powder, 1,2-cyclohexanedicarboxylic anhydride (HHPA), 2-ethyl-4-methylimidazole (2,4-EMI) and 3-chloroperoxybenzoic (MCPBA, 75%) were purchased from Sigma-Aldrich and used directly without further purification.

**Preparation of Bis-furan di-epoxide (BFDE)**


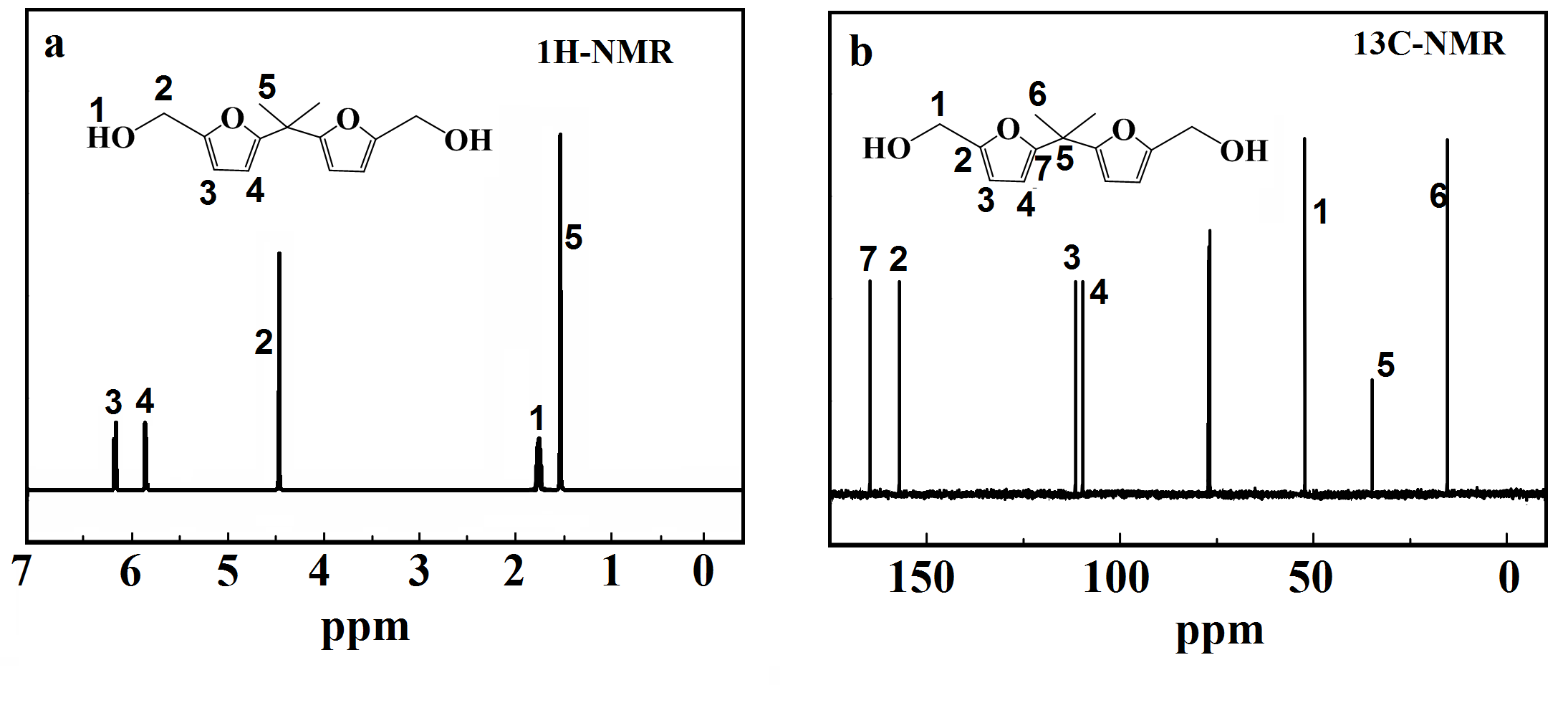


**Figure S1.** ^1^H-NMR spectrum (a) and ^13^C-NMR spectrum (b) of BFDM

Bis-furan di-epoxide (BFDE) was conducted following the literatures^S2^ with some improvements. The first step is the synthesis of bis-furan di-methanol (BFDM): 100g FCA, 1.0 M dry HCl with methanol (500 mL) which were added into 1L three necked round-bottom flask with a reflux condenser and mixed was heated to reflux for 3 h. Then, system was cooled to 0°C, 100 mL 98% H_2_SO_4_ was slowly added over a period of 30 min before the mixture was heated to 80 °C and maintained at this temperature for another 24 h. The system was cooled to room temperature and filtered, washed by 5% sodium bicarbonate and dried. The system was purified by flash chromatography (ethyl acetate:hexane =1:5) on silica to obtain 33.5g yellow solid. The solid and 10g LiAlH_4_ were added in to 500mL anhydrous THF under nitrogen atmosphere. The mixture was then stirred at room temperature for 24 h. The organic layer was quenched by concentrated sodium sulfate solution. The final crude product was then purified by flash chromatography (ethyl acetate:hexane=1:1) on silica to obtain 25.6g BFDM (yield ~95%). BFDM: ^1^H NMR (CD_3_Cl, d ppm) 6.20 (d. 2H), 5.95 (d, 2H), 4.55 (s, 4H), 2.15 (m, 2H), 1.70-1.60 (s, 6H); ^13^C (CD_3_Cl, d ppm) 156.80, 152.60, 108.50, 105.90, 53.40, 34.30, 19.20.


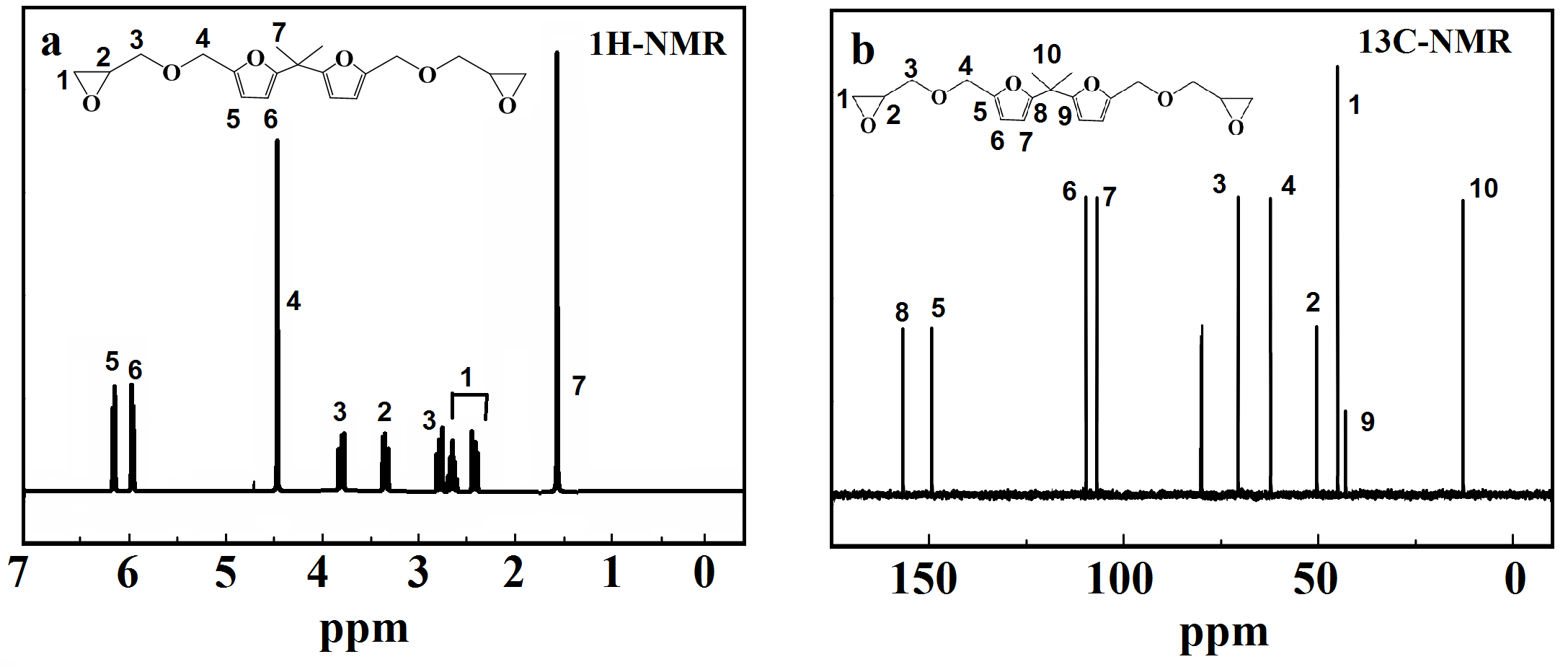


**Figure S2.** ^1^H-NMR spectrum (a) and ^13^C-NMR spectrum (b) of BFDE

The second-step is the synthesis of bis-furan di-epoxide (BFDE) ^S2^: epichlorohydrin (45 g) and tetrabutylammonium hydrogen sulfate (1.5 g) were added into a 500 mL threenecked round-bottom flask. The system temperature was kept in an ice-water bath at 0 C. the BFDM (25 g) was dropped into above the solution over a period of 30 min. Then NaOH aqueous (60mL, 50 wt%) was added into the mixture over a period of 60 min and controlled the system temperature was not more than 10 C. After 4 h, crude product was washed by water three times, and the organic layer was collected and dried by magnesium sulfate anhydrous for 24 h, and the solvent was removed using by a rotary evaporator. The product was obtained by chromatography on a silica gel column with mixed solvent of ethyl acetate/hexane (3:1) by flash chromatography (33.1g, ~89%). BFDE: ^1^H NMR (CD_3_Cl, d ppm) 6.25 (m, 2H), 6.05 (m, 2H), 4.50 (s, 4H), 3.80 (m, 2H), 3.28 (m, 2H), 2.95 (m, 2H), 2.50-2.65 (m, 2H), 1.62 (s, 6H); ^13^C (CD_3_Cl, d ppm) 160.20, 151.50, 109.50, 104.70, 71. 0, 64.90, 51.50, 47.60, 45.80, 25.40.

**Preparation of GO sheets**

Graphite oxide was fabricated by completely oxidizing graphite flakes based on a modified Hummer’s method in a solution of nitric acid, sulfuric acid and potassium chlorate for about 72 h ^S3^. The final powder was field and dried in a vacuum oven at 60°C for 24 h. Then the powder was dispersed into distilled water with strong stirring and ultrasonication for about 24 h to obtain the exfoliated GO sheets.


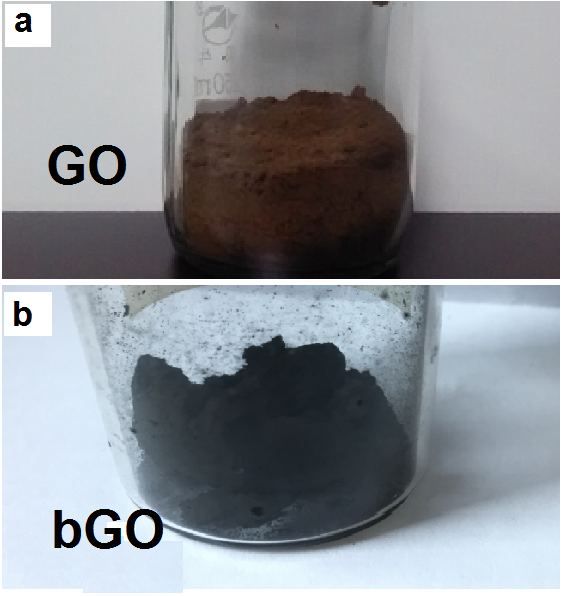


**Figure S3.** The change of color before and after modification of GO.


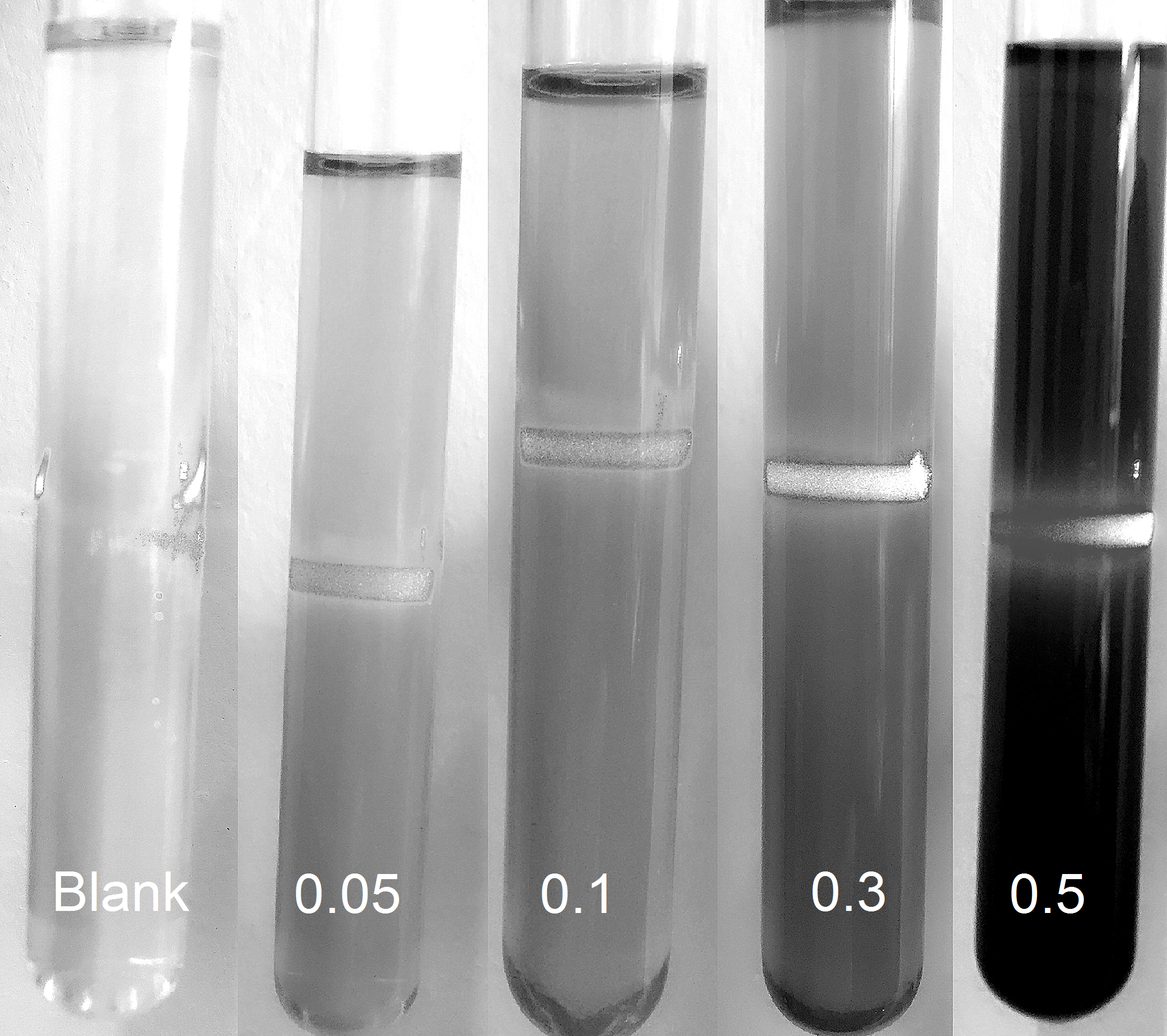


**Figure S4.** The colloidal solutions of bGO/epoxy mixtures and Tyndall effect.

**Reference.**

S1. Cho, J. K.; Lee, J. S.; ; Jeong, J.; Kim, B.; Kim, B.; Kim, S. Y. Synthesis of carbohydrate biomass-based furanic compounds bearing epoxide end group(s) and evaluation of their feasibility as adhesives. *J. Adhe. Sci. Technol.,***2013**, 27, 2127-2138.

S2. J. Ding, O. Rahman, Q. Wang, W. Peng and H. Yu, *ACS Sustainable Chem. Eng.* **2017**, 5, 7792-7799.

S3. Hu, F.; Scala, J. J. L.; Sadler, J. M.; Palmese, G. R. Synthesis and characterization of thermosetting furan-based epoxy systems. *Macromolecules,* **2014**, *47*(10), 3332-3342.

1. ^*^ [↑](#footnote-ref-2)
